# The microbiome and its impact on the epigenome in cancer

**DOI:** 10.3389/freae.2026.1857307

**Published:** 2026-06-17

**Authors:** Akhil Kapoor, Rohit Kumar, Anuj Gupta, Bipinesh Sansar, Bal Krishna Mishra, Mahesh Chaudhary, Archi Rungta, Gopalakrishna Kallapura, Kumar Prabhash

**Affiliations:** 1 Departments of Medical Oncology, Homi Bhabha Cancer Hospital & Mahamana Pandit Madan Mohan Malaviya Cancer Centre, Tata Memorial Centre, Homi Bhabha National Institute (HBNI), Varanasi, Uttar Pradesh, India; 2 SVB Cancer Research Laboratory, Homi Bhabha Cancer Hospital & Mahamana Pandit Madan Mohan Malaviya Cancer Centre, Tata Memorial Centre, Homi Bhabha National Institute (HBNI), Varanasi, Uttar Pradesh, India; 3 Microbiome Domain, Leucine Rich Bio Pvt Ltd., Bengaluru, Karnataka, India; 4 Department of Medical Oncology, Tata Memorial Hospital, Tata Memorial Centre, Homi Bhabha National Institute (HBNI), Mumbai, Maharashtra, India

**Keywords:** cancer, DNA methylation, dysbiosis, epigenome, histone modification, microbial metabolites, microbiome, oncobiome

## Abstract

The complex interplay between the human microbiome and epigenetic regulation represents one of the most rapidly evolving frontiers in cancer biology. The trillions of microorganisms residing in and on the human body collectively termed the microbiota may influence the host gene expression through epigenetic mechanisms, including DNA methylation, histone modification, and non-coding RNA regulation. In cancer patients, is associated with disruption of the normal epigenetic landscape, contributing to oncogenesis, tumor progression, immune evasion, and therapeutic resistance. This review synthesizes current evidence on how specific microbial metabolites such as short-chain fatty acids, secondary bile acids, and folate derivatives modulate epigenetic marks in tumor cells and the tumor microenvironment. We further discuss the emerging concept of the “oncobiome” and examine how microbiome-epigenome crosstalk influences cancer risk, progression, and response to therapy including chemotherapy, immunotherapy, and epigenetic drugs. Finally, we explore the translational potential of microbiome modulation as an adjunct strategy in cancer epigenomics, highlighting key research gaps and future directions.

## 1 Introduction

Cancer is fundamentally a disease of aberrant gene regulation, in which both genetic mutations and epigenetic alterations drive malignant transformation and progression (Hanahan and Weinberg, 2011). Over the past 2 decades, epigenomics has emerged as a critical dimension of cancer biology, revealing how reversible chemical modifications to DNA and associated histone proteins alter gene expression without changing the underlying nucleotide sequence (Jones and Baylin, 2007). Simultaneously, advances in metagenomic sequencing have illuminated the extraordinary complexity and functional diversity of the human microbiota. The vast community of bacteria, viruses, fungi, archaea, and protozoa affects various body compartments (Sender et al., 2016).

The adult human body harbors approximately 3.8 × 10^13 microbial cells, a number roughly equal to the total count of human cells, with the gastrointestinal tract housing the greatest density and diversity (L et al., 2016). These organisms are not merely passive commensals; they actively participate in metabolic, immunologic, and regulatory functions that are essential for host homeostasis. Their capacity to produce bioactive molecules capable of crossing epithelial barriers and entering systemic circulation positions them as potent modulators of host gene expression, including epigenetic regulation in distant tissues (Postler and Ghosh, 2017).

Compelling evidence now indicates that the microbiome has been implicated in multiple hallmarks, including sustaining proliferative signaling, evading growth suppressors, activating invasion and metastasis, enabling replicative immortality, and resisting cell death (Schwabe and Jobin, 2013). Crucially, many of these influences operate through epigenetic mechanisms. Microbial metabolites such as butyrate, propionate, secondary bile acids, and methyl-group donors serve as substrates or inhibitors of epigenetic enzymes, directly reshaping the cancer epigenome (Schroeder and Backhed, 2016). Moreover, in cancer patients receiving treatment, shifts in microbiome composition can alter drug metabolism and modify epigenetic responses to therapy, with meaningful consequences for outcomes (Zitvogel et al., 2018).

This review provides a comprehensive synthesis of the mechanistic links between the microbiome and the epigenome in cancer, with particular focus on clinical relevance. We examine key microbial taxa and metabolites implicated in epigenetic dysregulation, discuss the emerging concept of the oncobiome, and evaluate the translational prospects of targeting the microbiome-epigenome axis in oncology (Figure 1).

**FIGURE 1:**
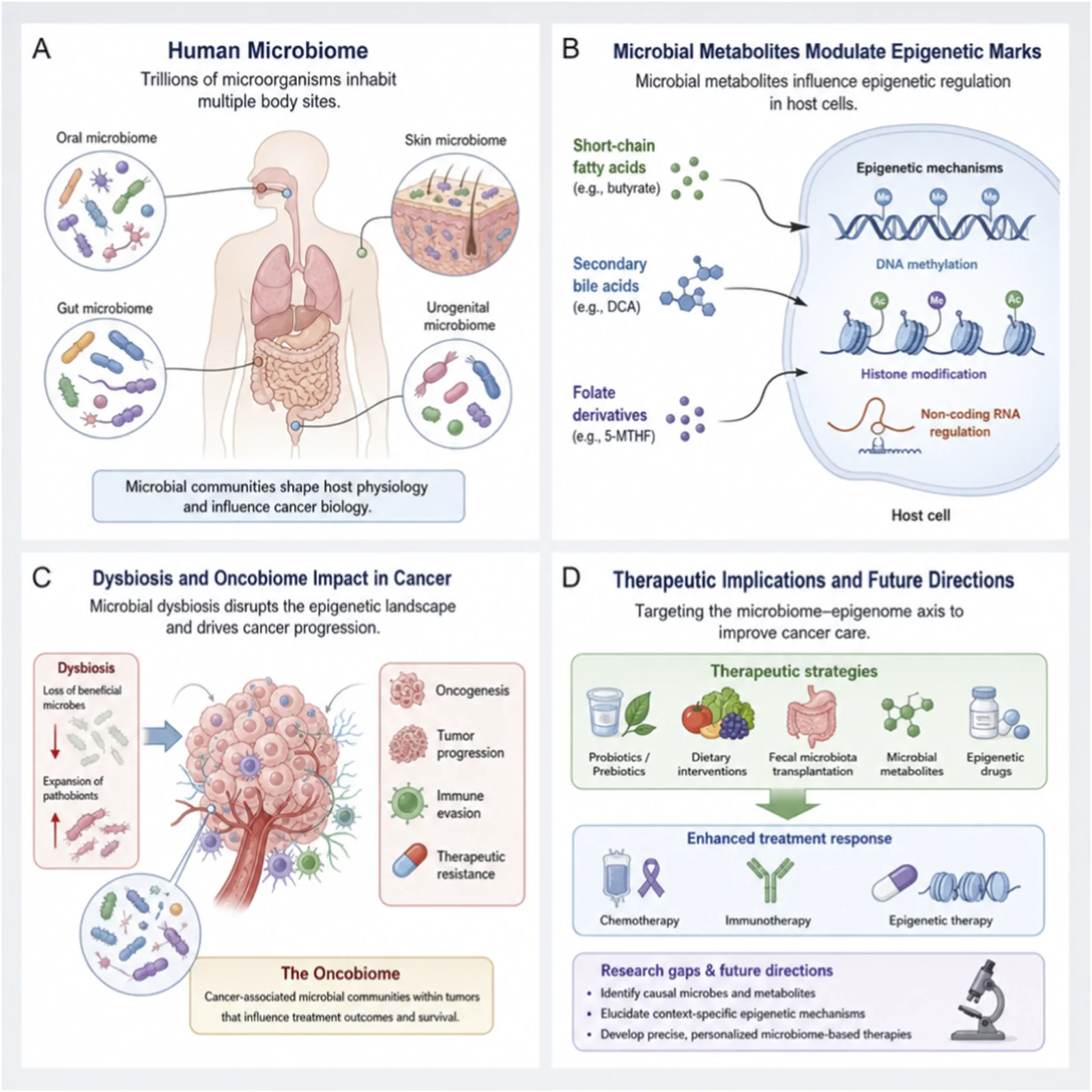
The microbiome–epigenome axis in cancer biology. **(A)** The human microbiota comprises trillions of microorganisms inhabiting multiple body sites, including the oral cavity, skin, gut, and urogenital tract, where they influence host physiology and cancer microenvironment; **(B)** Microbial-derived metabolites, such as short-chain fatty acids (e.g., butyrate), secondary bile acids, and folate derivatives, modulate host epigenetic mechanisms, including DNA methylation, histone modification (acetylation, methylation), and non-coding RNA regulation; **(C)** Dysbiosis in the oncobiome alter epigenetic homeostasis, contributing to oncogenesis, tumor progression, immune evasion, and therapeutic resistance; **(D)** Emerging therapeutic strategies targeting the microbiome–epigenome axis including probiotics or prebiotics, dietary interventions, fecal microbiota transplantation, microbial metabolites, and epigenetic drugs may enhance responses to chemotherapy, immunotherapy, and epigenetic therapy. The figure was generated using BioRender.com.

## 2 Epigenetic mechanisms in cancer: a brief overview

Epigenetics encompasses heritable changes in gene expression that occur without alterations in the DNA sequence itself (Baylin and Jones, 2016). In cancer, three principal layers of epigenetic regulation are perturbed: DNA methylation, histone modification, and non-coding RNA-mediated gene silencing (Figure 1). Epigenetics encompasses heritable changes in gene expression that occur without alterations in the DNA sequence itself. A central question in oncology is whether these epigenetic alterations act as primary causal drivers of tumorigenesis or as secondary consequences of the transformed cellular state. Current evidence indicates they operate as both. As primary “epi-drivers,” early epigenetic events can precede and actually provoke genetic changes, such as when the silencing of critical DNA repair genes leads to the rapid accumulation of mutations (Sawan et al., 2008). These epimutations can inappropriately silence tumor suppressor genes or activate oncogenes prior to any genetic mutation, initiating malignant clonal expansion and playing a dynamic role in driving metastatic phenotypes (Chatterjee et al., 2018). Widespread alterations, such as the CpG island methylator phenotype (CIMP) and long-range epigenetic silencing (LRES), further favor cancer progression by coordinately repressing tumor suppressor networks across multiple genomic regions (Yates and Boeva, 2022; Javierre et al., 2011). Conversely, as secondary consequences, or “epi-passengers,” the altered metabolic landscape of a developing tumor can dysregulate the activity of epigenetic modifier enzymes, resulting in a metabolic recoding of the epigenome (Wang and Lei, 2018). Furthermore, structural chromatin remodelers and pioneering transcription factors are often hijacked during tumorigenesis to alter chromatin accessibility, creating a reinforcing feedback loop that accelerates tumor progression and phenotypic heterogeneity (Kgatle et al., 2025). In cancer, three principal layers of this epigenetic regulation are perturbed: DNA methylation, histone modification, and non-coding RNA-mediated gene silencing.

### 2.1 DNA methylation

DNA methylation involves the covalent addition of a methyl group to the 5-carbon position of cytosine residues, predominantly within CpG dinucleotides, catalyzed by DNA methyltransferases (DNMTs) (Este and ller, 2008). In normal tissues, CpG islands which are clusters of CpG dinucleotides and found in promoter regions are generally unmethylated, maintaining transcriptional accessibility of tumor suppressor genes. In cancer, widespread hypomethylation of repetitive elements and focal hypermethylation of CpG island promoters are hallmark findings, resulting in genomic instability and silencing of critical regulatory genes including CDH1, MLH1, CDKN2A, and BRCA1 (Herman and Baylin, 2003).

### 2.2 Histone modifications

Histones, the protein scaffolds around which DNA is wound, undergo a diverse array of post-translational modifications including acetylation, methylation, phosphorylation, ubiquitination, and SUMOylation (Bannister and Kouzarides, 2011). These modifications, orchestrated by writer, eraser, and reader enzymes, determine chromatin compaction and transcriptional activity. In cancer, dysregulation of histone deacetylases (HDACs) and histone acetyltransferases (HATs) frequently results in transcriptional silencing of tumor suppressors and activation of oncogenes. Similarly, aberrant histone methylation especially at H3K4, H3K27, and H3K9 is a characteristic feature of multiple malignancies (Chi et al., 2010).

### 2.3 Non-coding RNAs

Non-coding RNAs (ncRNAs), including microRNAs (miRNAs), long non-coding RNAs (lncRNAs), and circular RNAs (circRNAs), constitute a third layer of epigenetic regulation. They modulate gene expression post-transcriptionally, regulate chromatin-modifying complexes, and serve as sponges for other regulatory molecules (Slack and Chinnaiyan, 2019). Dysregulation of ncRNA expression profiles is ubiquitous in cancer and contributes to virtually all stages of tumor development. The microbiome has been shown to influence ncRNA expression, adding another dimension to microbiome-epigenome crosstalk (Duttagupta et al., 2011).

## 3 The human microbiome in oncology

### 3.1 The concept of the oncobiome

The term “oncobiome” has been proposed to describe the specific microbial configurations associated with cancer initiation, progression, or protection (Nejman et al., 2020). Microbial dysbiosis characterized by perturbation in the composition, diversity, or function of the microbiome is a consistent finding across multiple cancer types. Whether dysbiosis is a cause or consequence of malignancy remains a matter of active investigation; evidence increasingly supports bidirectional relationships (Garrett, 2019) (Figure 1).

Landmark studies using 16 S rRNA sequencing and shotgun metagenomics have characterized disease-specific microbial signatures in colorectal cancer (CRC), breast cancer, lung cancer, hepatocellular carcinoma, and pancreatic ductal adenocarcinoma, among others (Bars-Cortina et al., 2024; Mas-Lloret et al., 2020). In CRC, for example, *Fusobacterium nucleatum* is enriched and correlates with worse prognosis and resistance to chemotherapy, partly through epigenetic mechanisms (Rubinstein et al., 2013). In breast cancer, alterations in the breast tissue and gut microbiome are associated with estrogen metabolism and DNA methylation changes (Xuan et al., 2014). Mechanistically, *F. nucleatum* colonization is strongly associated with the CpG island methylator phenotype (CIMP), which drives the DNA promoter hypermethylation and subsequent silencing of tumor suppressor and mismatch repair genes such as MLH1 (Nosho et al., 2016). Furthermore, this pathogen alters the post-transcriptional epigenetic landscape by upregulating oncogenic microRNAs, specifically miR-21 and miR-155, which hyperactivate inflammatory and Wnt/β-catenin signaling pathways (Bhatt et al., 2017). To promote therapeutic resistance to agents like 5-fluorouracil, *F. nucleatum* also utilizes miRNA-driven epigenetic silencing of apoptotic pathways while upregulating autophagy-related genes (Yu et al., 2017). However, these disease-associated patterns must be interpreted cautiously. Current studies often exhibit significant heterogeneity, and it remains a challenge to definitively separate causal microbial drivers from correlative changes influenced by confounding factors such as patient diet, environmental exposures, and prior antibiotic use.

### 3.2 Routes of microbial influence on distant tissues

While the gut microbiota exerts the most studied influence, microbial effects on the epigenome can be transmitted through multiple routes. First, the circulation of microbial metabolites via the portal and systemic vasculature allows compounds like short-chain fatty acids to function as systemic histone deacetylase (HDAC) inhibitors (Donohoe et al., 2012), while folate derivatives supply methyl groups for DNA methylation (Stover, 2009). Second, the translocation of microbial products such as lipopolysaccharide (LPS), bacterial DNA, and peptidoglycans engages host pattern recognition receptors (e.g., TLR4) to trigger intracellular signaling cascades that recruit DNA methyltransferases (DNMTs) to target gene promoters (Yu and Schwabe, 2017). Third, modulation of the immune system by the microbiota shapes the epigenetic landscape of immune and stromal cells in the tumor microenvironment through cytokine-driven pathways, such as IL-6/STAT3 signaling, that promote aberrant chromatin remodeling (Thaiss et al., 2016; Maekit et al., 2006). The “gut-liver axis,” “gut-lung axis,” and other inter-organ microbiome-mediated pathways have been delineated as conduits for microbial epigenetic influence in cancers arising outside the gastrointestinal tract (Dhar et al., 2020).

## 4 Microbial metabolites as epigenetic modulators in cancer

### 4.1 Short-chain fatty acids

Short-chain fatty acids (SCFAs) such as butyrate, propionate, and acetate are the principal fermentation products of dietary fiber by colonic anaerobes including *Faecalibacterium prausnitzii*, *Roseburia* species, and *Bacteroides* species (Koh et al., 2016). Among SCFAs, butyrate is the most extensively studied epigenetic regulator. It is a potent inhibitor of class I and class IIa HDACs, elevating histone acetylation at lysine residues and thereby increasing chromatin accessibility (Donohoe et al., 2012). In normal colonocytes, butyrate maintains cellular homeostasis and suppresses inflammation; however, in the context of dysbiosis leading to butyrate depletion, reduced HDAC inhibition contributes to epigenetic silencing of tumor suppressors and malignant transformation (Louis et al., 2014).

Propionate, another SCFA, has been shown to inhibit DNMTs as well as HDACs, contributing to demethylation of tumor suppressor gene promoters. Acetate serves as a substrate for histone acetyltransferases through its conversion to acetyl-CoA, linking microbial fermentation directly to the histone acetylome (Waldecker et al., 2008). Reduced SCFA production due to fiber-poor diets or antibiotic-mediated dysbiosis is considered a plausible epigenetic pathway linking lifestyle factors to colorectal carcinogenesis (Zackular et al., 2013) (Table 1).

**TABLE 1:** Microbial metabolites and products as epigenetic modulators in cancer.

Metabolite/Product	Producing organism(s)	Epigenetic target/Mechanism	Epigenetic effect in cancer	Cancer Type(s) [Ref]
Butyrate (SCFA)	*Faecalibacterium prausnitzii, Roseburia* spp.	HDAC inhibition (Class I, IIa)	↑ Histone acetylation (H3K9ac, H3K27ac); restored tumor suppressor expression	Colorectal, pancreatic (Donohoe et al., 2012; Louis et al., 2014; Waldecker et al., 2008)
Propionate (SCFA)	*Bacteroides* spp., *Akkermansia muciniphila*	DNMT inhibition; HDAC inhibition	DNA hypomethylation of tumor suppressor promoters	Colorectal (Waldecker et al., 2008)
Acetate (SCFA)	Various anaerobes	Histone acetyltransferase substrate (acetyl-CoA)	↑ Global histone acetylation	Colorectal, lung (Waldecker et al., 2008; Tsay et al., 2018)
Secondary bile acids (DCA, LCA)	*Clostridium* cluster XIVa, Clostridiales	↑ DNMT3A/3 B activity; ↑ EZH2	Promoter hypermethylation (SFRP1, DKK1); ↑ H3K27me3	Colorectal, hepatocellular (Yu et al., 2017; Ridlon et al., 2014; Ajouz et al., 2014)
Folate/B vitamins	*Lactobacillus, Bifidobacterium, Enterococcus* spp.	SAM availability for DNMT/HMT	Altered DNA and histone methylation; loss of imprinting	Colorectal, breast (Stover, 2009; Duthie, 2011)
Colibactin	pks + *Escherichia coli*	DNA DSB-triggered DNMT1 recruitment	Aberrant methylation at repair sites; genomic instability	Colorectal (Nougayrede et al., 2006)
*Bacteroides fragilis* toxin (BFT)	Enterotoxigenic *B. fragilis* (ETBF)	NF-κB/STAT3 → HDAC activation	E-cadherin promoter hypermethylation; H3K27me3 ↑	Colorectal (Wu et al., 2003)
CagA protein	*Helicobacter pylori*	Direct DNMT interaction; IL-6/STAT3	Promoter hypermethylation (MLH1, CDH1, CDKN2A)	Gastric, esophageal (Maekit et al., 2006)
Bacterial small RNAs (OMVs)	Multiple Gram-negative bacteria	miRNA mimicry/Argonaute interaction	Post-transcriptional silencing of tumor suppressors	Multiple types (Bhatt et al., 2017; Koeppen et al., 2016)

Abbreviations: SCFA, short-chain fatty acid; HDAC, histone deacetylase; DNMT, DNA methyltransferase; EZH2, enhancer of zeste homologue 2; SAM, S-adenosylmethionine; DCA, deoxycholic acid; LCA, lithocholic acid; BFT, *bacteroides fragilis* toxin; DSB, double-strand break; OMV, outer membrane vesicle; MMR, mismatch repair; HMT, histone methyltransferase; CRC, colorectal cancer; HCC, hepatocellular carcinoma; PDAC, pancreatic ductal adenocarcinoma.

### 4.2 Secondary bile acids

Primary bile acids synthesized in the liver are converted to secondary bile acids, i.e., deoxycholic acid (DCA) and lithocholic acid (LCA) by intestinal bacteria, particularly members of *Clostridium* cluster XIVa (Ridlon et al., 2014). Secondary bile acids are established tumor promoters, especially in CRC and hepatocellular carcinoma. Their epigenetic effects include activation of DNMT3A and DNMT3B, leading to promoter hypermethylation of key tumor suppressors such as SFRP1 and DKK1 (Ajouz et al., 2014). DCA has also been shown to induce aberrant histone methylation patterns by upregulating EZH2, promoting H3K27 trimethylation and silencing of differentiation genes (Ma et al., 2025; Verma et al., 2022; Xu et al., 2020).

### 4.3 Folate and one-carbon metabolism

Several bacteria including *Lactobacillus, Bifidobacterium*, and *Enterococcus* species synthesize folate and other B vitamins critical for one-carbon metabolism. This biochemical pathway generates S-adenosylmethionine (SAM), the universal methyl donor for DNA and histone methylation reactions (Stover, 2009). Microbial dysbiosis depleting folate-producing bacteria can reduce SAM availability, causing widespread DNA hypomethylation and loss of imprinting in colonocytes (Duthie, 2011). The balance of microbial one-carbon metabolism plays a nuanced role in shaping the cancer methylome.

### 4.4 Microbial toxins and DNA methylation

Colibactin, produced by pks + *Escherichia coli* strains frequently found in CRC patients, induces DNA double-strand breaks that trigger epigenetic stress responses including activation of DNMT1 at repair sites, creating a link between microbial genotoxicity and aberrant methylation (Nougayrede et al., 2006). *Bacteroides fragilis* toxin (BFT) secreted by enterotoxigenic *Bacteroides fragilis* (ETBF) activates NF-κB and STAT3 signaling cascades, downstream of which HDAC activation and promoter hypermethylation of E-cadherin occurs (Wu et al., 2003). *Helicobacter pylori* CagA protein directly interacts with DNMT enzymes, driving widespread methylation of gastric tumor suppressor gene promoters (Maekit et al., 2006).

## 5 Microbiome, immune epigenetics, and the tumor microenvironment

Beyond direct effects on tumor cells, the microbiome profoundly shapes the epigenetic state of immune cells within the tumor microenvironment (TME), with far-reaching consequences for anti-tumor immunity and cancer progression (Gopalakrishnan et al., 2018).

### 5.1 Epigenetic regulation of tumor-infiltrating lymphocytes

Butyrate and other SCFAs regulate the differentiation and function of regulatory T cells (Tregs) through HDAC inhibition, promoting Foxp3 gene expression via histone acetylation at the Foxp3 locus (Furusawa et al., 2013). Elevated Treg infiltration in the TME is associated with immune suppression and poor prognosis. Conversely, microbial signals that activate pattern recognition receptors on dendritic cells induce epigenetic reprogramming toward an immunostimulatory phenotype, enhancing CD8^+^ T cell cytotoxicity against tumor cells (Dzutsev et al., 2015).

### 5.2 Trained immunity and epigenetic memory

The concept of trained immunity whereby innate immune cells undergo epigenetic reprogramming after an initial stimulus has been increasingly linked to microbial signals (Netea et al., 2016). Microbial metabolites and cell wall components can induce H3K4 monomethylation and H3K27 acetylation at enhancers of innate immune genes in monocytes and macrophages, creating durable epigenetic memory that shapes anti-tumor immune responses. In cancer patients with dysbiosis, this program may be skewed toward pro-inflammatory or immunosuppressive phenotypes that promote tumor progression (Arts et al., 2018).

### 5.3 miRNA regulation by the microbiome in the TME

*Fusobacterium nucleatum* upregulates miR-21 and miR-155 in CRC cells, promoting Wnt/β-catenin signaling and inflammatory pathways while downregulating tumor suppressor miRNAs (Bhatt et al., 2017). Bacterially derived outer membrane vesicles (OMVs) can deliver bacterial small RNAs directly into host cells, where they interact with Argonaute complexes to modulate gene expression in the TME (Koeppen et al., 2016).

## 6 Cancer type-specific microbiome-epigenome interactions

The following section summarizes cancer type-specific findings; Table 2 provides a comparative overview across malignancies.

**TABLE 2:** Cancer type-specific microbiome alterations and epigenetic consequences.

Cancer type	Enriched taxa	Depleted taxa	Key epigenetic findings	Clinical significance	References
Colorectal (CRC)	*Fusobacterium nucleatum*, ETBF, pks + *E. coli*, *P. anaerobius*	Depletion of *Faecalibacterium prausnitzii, Roseburia*	CIMP (MLH1, CDKN2A, SFRP1 methylation); ↓ H3K9ac due to SCFA loss	Worse prognosis; 5-FU and oxaliplatin resistance	Rubinstein et al. (2013), Nosho et al. (2016), Brennan and Garrett (2019)
Breast	*Fusobacterium* spp., *Staphylococcus* in tumor tissue	↓ Folate-producing bacteria; ↑ beta-glucuronidase bacteria	Altered methylation at estrogen-responsive loci; ↑ circulating estrogen driving DNMT expression	ER + subtype enrichment; altered tamoxifen response	Xuan et al. (2014), Kwa et al. (2016), Goedert et al. (2015)
Hepatocellular (HCC)	*Klebsiella pneumoniae*, *Proteobacteria* enriched	↓ *Akkermansia muciniphila*, Lachnospiraceae	LPS via portal vein → TLR4 → DNMT3B ↑; RASSF1A, p16 hypermethylation; secondary BA → EZH2 ↑	Tumor development in cirrhosis; sorafenib resistance	Yu and Schwabe (2017), Ponziani et al. (2019)
Gastric	*Helicobacter pylori* (CagA+)	↓ Microbial diversity	CpG island hypermethylation of MLH1, CDH1, RUNX3 via DNMT upregulation; epigenetic field defect	Gastric carcinogenesis; cisplatin resistance	Maekit et al. (2006), Gopalakrishnan et al. (2018)
Lung	*Veillonella, Prevotella* enriched in airways	↓ Diversity in tumor-associated microbiome	Acetate/propionate modulate histone acetylation in bronchial epithelium; PI3K pathway activation	Immunotherapy response variation	Tsay et al. (2018)
Pancreatic (PDAC)	Gammaproteobacteria intratumoral	↓ Gut eubiosis	Intratumoral bacteria alter histone methylation; metabolize gemcitabine	Chemotherapy resistance	Geller et al. (2017)

Abbreviations: CRC, colorectal cancer; CIMP, CpG island methylator phenotype; HCC, hepatocellular carcinoma; PDAC, pancreatic ductal adenocarcinoma; ETBF, enterotoxigenic *Bacteroides fragilis*; ER+, estrogen receptor-positive; BA, bile acids; TLR4, toll-like receptor 4; LPS, lipopolysaccharide; MMR, mismatch repair; MSI-H, microsatellite instability-high; NSCLC, non-small cell lung cancer.

### 6.1 Colorectal cancer

CRC provides the most comprehensive paradigm for microbiome-epigenome crosstalk in oncology. The CRC-associated microbiota is characterized by enrichment of *F. nucleatum*, ETBF, pks + *Escherichia coli*, and *Peptostreptococcus anaerobius*, and depletion of butyrate-producing bacteria (Brennan and Garrett, 2019). The CpG island methylator phenotype (CIMP) accounts for approximately 20% of CRC cases. Several studies have reported significant association of *F. nucleatum* with high-CIMP CRC (Nosho et al., 2016; Tahara et al., 2014; Mima et al., 2015; Mima et al., 2016; Ito et al., 2015). However, the mechanistic role of *F. nucleatum* in CIMP remains unconfirmed, and its association with CRC may be correlative rather than causative.

### 6.2 Breast cancer

Breast cancer is associated with alterations in both the gut microbiome and the breast tissue microbiota. Gut dysbiosis, particularly an overrepresentation of beta-glucuronidase-producing bacteria, increases the deconjugation of estrogen glucuronides. This elevates circulating estrogen levels, which strongly drives ER + breast cancer (Kwa et al., 2016). Mechanistically, this sustained estrogenic signaling can upregulate DNA methyltransferases (DNMTs), driving aberrant DNA methylation at estrogen-responsive loci (Goedert et al., 2015). Furthermore, dysbiosis-driven epigenetic changes in breast cancer have been linked to the hypermethylation of tumor suppressor microRNAs, such as miR-125b1, which correlates with a loss of CTCF boundary-element binding and the subsequent accumulation of repressive histone marks like H3K9me3 and H3K27me3 (Soto-Reyes et al., 2012).

### 6.3 Hepatocellular carcinoma

In HCC, gut-derived LPS and secondary bile acids reach the liver via the portal circulation, heavily impacting the epigenetic landscape. LPS engages Toll-like receptor 4 (TLR4), triggering intracellular inflammatory cascades that specifically upregulate DNMT3B. This leads to the promoter hypermethylation and silencing of critical tumor suppressors, including RASSF1A and p16/CDKN2A (Yu and Schwabe, 2017). Concurrently, microbe-derived secondary bile acids promote aberrant chromatin remodeling by upregulating EZH2, the catalytic subunit of the Polycomb Repressive Complex 2 (PRC2), which deposits repressive H3K27me3 marks to silence differentiation pathways (Ma et al., 2025). Cirrhosis-associated dysbiosis, characterized by the depletion of *Akkermansia muciniphila* and enrichment of Proteobacteria, thus creates an epigenetically permissive environment for hepatocellular transformation by continuously fueling these altered methylation and histone modification networks (Ponziani et al., 2019).

### 6.4 Lung and pancreatic cancers

In lung cancer, the airway microbiota significantly impacts the tumor microenvironment. Local dysbiosis, characterized by an overall decrease in microbial diversity and the enrichment of taxa such as *Veillonella* and *Prevotella*, leads to the production of short-chain fatty acids like acetate and propionate. These metabolites modulate global histone acetylation in bronchial epithelial cells, which epigenetically drives the transcriptional upregulation of the PI3K signaling pathway, thereby promoting cell proliferation and tumor progression (Tsay et al., 2018). These microbial influences act alongside prevalent epigenetic aberrations in lung tumorigenesis, such as the frequent inactivation of the p16/CDKN2A tumor suppressor (Tam et al., 2013) and the hypomethylation-driven overexpression of the FEN1 DNA repair gene, which contributes to genomic instability (Sato et al., 2003). Beyond local airway interactions, the composition of the systemic gut microbiota also plays a critical role in non-small cell lung cancer (NSCLC) by determining the variation in patient response to PD-1/PD-L1 immune checkpoint inhibitor therapies (Routy et al., 2018).

### 6.5 Pancreatic ductal adenocarcinoma (PDAC)

Pancreatic tumors harbor a distinct intracellular microbiota, notably enriched in Gammaproteobacteria. These intratumoral bacteria profoundly influence the tumor microenvironment by directly metabolizing the chemotherapeutic agent gemcitabine into its inactive form, conferring severe drug resistance (Geller et al., 2017; Routy et al., 2018). Concurrently, this local microbial presence, combined with decreased gut eubiosis, alters global histone methylation networks, further entrenching the aggressive and chemoresistant epigenetic phenotype of PDAC. The dysregulated epigenetic landscape of pancreatic cancer is additionally fueled by the upregulation of chromatin-associated architectural proteins like HMGA1, which further modulate oncogenic transcription (Sgarra et al., 2004), alongside the promoter hypomethylation of genes such as FEN1 that drive error-prone DNA repair pathways (Iacobuzio-Donahue et al., 2003).

## 7 Microbiome-epigenome axis and cancer therapy

### 7.1 Influence on epigenetic drug efficacy

Epigenetic therapies including DNMT inhibitors (azacitidine, decitabine) and HDAC inhibitors (vorinostat, romidepsin) are approved for hematologic malignancies and are under investigation for solid tumors (Heinhuis et al., 2019). The microbiome influences the pharmacokinetics and pharmacodynamics of these agents. Gut bacteria can metabolize DNMT inhibitors, reducing bioavailability, while SCFA-producing bacteria may synergize with HDAC inhibitors by providing endogenous HDAC inhibition (Alexander et al., 2017). However, the clinical validation of these microbiome-drug interactions remains in its early stages, and substantial inter-patient variability in baseline microbiome composition complicates the predictable modulation of epigenetic drug efficacy (Table 3).

**TABLE 3:** Therapeutic interventions targeting the microbiome-epigenome axis in cancer.

Therapeutic agent/Intervention	Category	Epigenetic mechanism of action	Microbiome–epigenome interaction	Clinical context	References
HDAC Inhibitors (Vorinostat, Romidepsin)	Epigenetic therapy	Restores histone acetylation; reactivates tumor suppressors	Microbiome modulates bioavailability; SCFA-producing bacteria synergize with HDACi	Hematologic malignancies; emerging solid tumor data	Heinhuis et al. (2019), Alexander et al. (2017)
DNMT Inhibitors (Azacitidine, Decitabine)	Epigenetic therapy	Reverses aberrant promoter methylation; re-expresses silenced genes	Gut bacteria metabolize drug; dysbiosis may reduce efficacy	Myelodysplastic syndrome, AML; investigated in solid tumors	Heinhuis et al. (2019), Alexander et al. (2017)
PD-1/PD-L1 Checkpoint Inhibitors	Immunotherapy (ICI)	Blocks T cell exhaustion; restores anti-tumor immunity	*Faecalibacterium, Akkermansia*, Bifidobacterium → epigenetic remodeling of T cells → ↓ exhaustion markers	Melanoma, NSCLC, CRC (MSI-H); microbiome predicts response	Routy et al. (2018), Pitt et al. (2016)
Dietary Fiber Supplementation	Microbiome modulation	↑ SCFA-producing bacteria → butyrate/propionate → HDAC inhibition	Restores HDAC inhibitory activity; reverses tumor suppressor silencing in colonocytes	CRC prevention; adjunct to therapy	Vipperla and O'Keefe (2016)
Probiotics (*Lactobacillus, Bifidobacterium*)	Microbiome modulation	Restore eubiosis; produce folate and SCFAs	Altered DNA methylation patterns in colonic mucosa; potential normalization of cancer methylome	Adjunct cancer prevention; ICI sensitization under investigation	Vipperla and O'Keefe (2016), Baruch et al. (2021)
Fecal Microbiota Transplantation (FMT)	Microbiome modulation	Restores broad microbial diversity and metabolite production	Normalizes SCFA levels → histone acetylation; shifts T cell epigenetic state toward effector phenotype	ICI-refractory melanoma (Phase I/II); *C. difficile* approved	Baruch et al. (2021)
5-Fluorouracil (5-FU)	Chemotherapy	Antimetabolite; pyrimidine analogue	*F. nucleatum* → autophagy↑ via miRNA → resistance; dysbiosis alters drug metabolism	CRC, gastric; microbiome composition predicts toxicity	Yu et al. (2017)
Cisplatin/Gemcitabine	Chemotherapy	DNA crosslinking/nucleoside analogue	*H. pylori* → MLH1 methylation → MMR deficiency → resistance; intratumoral bacteria metabolize gemcitabine	Gastric (cisplatin), PDAC (gemcitabine)	Geller et al. (2017), Ushijima and Hattori (2012)

Abbreviations: DNMT, DNA methyltransferase inhibitor; HDAC, histone deacetylase inhibitor; ICI, immune checkpoint inhibitor; SCFA, short-chain fatty acid; FMT, fecal microbiota transplantation; AML, acute myeloid leukemia; CRC, colorectal cancer; PDAC, pancreatic ductal adenocarcinoma; NSCLC, non-small cell lung cancer; MMR, mismatch repair; MSI-H, microsatellite instability-high; HDACi, HDAC, inhibitor.

### 7.2 Immunotherapy and microbiome-epigenetic synergy

The relationship between the gut microbiome and immune checkpoint inhibitor (ICI) therapy is increasingly supported by emerging studies, with multiple studies demonstrating that microbiome composition predicts response to PD-1/PD-L1 and CTLA-4 blockade (Routy et al., 2018). Key responder-associated taxa produce SCFAs that remodel the epigenetic state of TME-resident T cells, promoting effector function and reducing exhaustion through HDAC inhibition at regulatory loci such as PD-1, LAG-3, and TIM-3 (Pitt et al., 2016).

### 7.3 Chemotherapy resistance and microbiome epigenetics

*Fusobacterium nucleatum* has been shown in preclinical cell line as well as patient derived xenograft animal model as resistance to 5-fluorouracil (5-FU) in CRC through upregulation of autophagy-related genes and miRNA-driven silencing of apoptotic pathways (Yu et al., 2017). *Helicobacter pylori* induces epigenetic silencing of MLH1 via promoter hypermethylation, impairing mismatch repair and contributing to cisplatin resistance in gastric cancer (Ushijima and Hattori, 2012).

## 8 Translational implications and future directions

### 8.1 Microbiome modulation as an epigenetic therapeutic strategy

The plasticity of both the microbiome and the epigenome offer unique therapeutic opportunities. Dietary interventions that increase fiber intake and promote SCFA-producing bacteria can restore HDAC inhibitory activity, reversing epigenetic silencing of tumor suppressors (Vipperla and O'Keefe, 2016). Fecal microbiota transplantation (FMT) is under clinical investigation as a strategy to restore eubiosis and normalize epigenetic dysregulation in cancer patients, particularly in conjunction with immunotherapy (Baruch et al., 2021).

### 8.2 Biomarker potential

Multi-omic integration of microbiome composition, microbial metabolomics, and tumor DNA methylation profiling may yield more accurate cancer risk stratification, early detection, and prognostic models (Bashiardes et al., 2017). Circulating microbial metabolites and microbiome-regulated miRNAs in blood represent minimally invasive biomarkers that could be integrated into liquid biopsy platforms.

### 8.3 Research gaps and challenges

Several key challenges limit progress in this field. Establishing causal directionality between microbiome changes and epigenetic alterations *in vivo* remains methodologically demanding, requiring germ-free animal models and rigorous longitudinal human studies (Dzutsev et al., 2017). Additionally, epigenome-microbiome interactions are highly tissue-specific and context-dependent, making generalizations across cancer types hazardous.

## 9 Conclusion

The microbiome-epigenome axis represents a fundamental and clinically significant dimension of cancer biology. Microbial communities influence every major epigenetic layer, e.g., DNA methylation, histone modification, and non-coding RNA regulation through a diverse repertoire of metabolites, toxins, and immune modulatory signals. In cancer patients, microbial dysbiosis reshapes the tumor epigenome, the immune epigenome, and the epigenetic response to therapy in ways that profoundly affect disease outcomes. The emerging recognition of the oncobiome as a dynamic epigenetic regulator opens new avenues for cancer prevention, biomarker discovery, and therapeutic innovation. Integrating microbiomics and epigenomics into multi-omic cancer research frameworks, supported by mechanistic *in vivo* studies and prospective clinical trials, will be essential to fully realize the translational potential of this nexus.
